# Electronic cigarette use and consumption patterns in medical university students

**DOI:** 10.3389/fpubh.2024.1403737

**Published:** 2024-09-30

**Authors:** Asli Gorek Dilektasli, Ozge Aydin Guclu, Arzu Ozpehlivan, Vahide Aslihan Durak, Izzet Gezmis, Anıl Ozgur, Burak Cinar, Ezgi Demirdogen, Nilufer Aylin Acet Ozturk, Guven Ozkaya, Funda Coskun, Ahmet Ursavas, Esra Uzaslan, Mehmet Karadag

**Affiliations:** ^1^Department of Pulmonary Medicine, Bursa Uludag University Faculty of Medicine, Bursa, Türkiye; ^2^Department of Emergency Medicine, Bursa Uludag University Faculty of Medicine, Bursa, Türkiye; ^3^Department of Biostatistics, Bursa Uludag University Faculty of Medicine, Bursa, Türkiye

**Keywords:** electronic cigarettes, electronic nicotine delivery systems, smoking, tobacco, young adults, medical students, perception, knowledge

## Abstract

**Background:**

A major public health hazard is youth e-cigarette use. Although new, e-cigarette health hazards are becoming well-known in the literature. E-cigarette sale restrictions and laws differ globally. In this cross-sectional study, we studied medical university students’ tobacco and e-cigarette use and characteristics in a country where sales and import of e-cigarettes are banned. The primary objective is to determine the prevalence of electronic cigarette use and understand consumption patterns among medical faculty students in this setting.

**Materials and methods:**

The questionnaire was sent using a web-based student information system. Sociodemographic features, tobacco and e-cigarette use, consumption patterns, and e-cigarette risk perceptions were covered in 54 questions.

**Results:**

The study comprised 1,054 students (48.7% male) aged 21.5 ± 2.6 years who completed the questionnaire. 37.7%, 20.9% and 23.6% have smoked cigarettes, e-cigarettes, or water pipes. Current cigarette smokers were 17.0%, e-cigarette users 4.0%, and water pipe smokers 4.5%. E-cigarette users were 52.3% dual smokers. The most common symptoms reported by e-cigarette users were cough (58.4%) and dyspnea (54.2%). Multivariable models showed that the male sex, greater monthly income, and a current smoker friend were independent risk factors for e-cigarette ever use, while the male sex, paternal current smoking, and close friends’ current smoking status were risk factors for dual use among medical trainees. Many medical students who used electronic cigarettes underestimated nicotine’s health hazards and harmful chemicals in e-cigarettes. Despite e-cigarette sales being prohibited in our country, 56.4% and 25.4% of e-cigarette users provided e-cigarettes from tobacco shops and through online sales, respectively.

**Conclusion:**

Medical university students use tobacco most often by smoking cigarettes. Despite medical university students being aware of the health hazards of e-cigarettes, the current use of electronic cigarettes is 4.0%. Male sex, greater monthly income, and having current smoker friends are independent risk factors for e-cigarette use, while paternal smoking is a risk factor for dual use among medical trainees. Although in the country, sales of e-cigarettes are banned, ever-use rates for e-cigarettes were remarkably high at 20.9%, and the ease of accessing e-cigarettes was striking.

## 1 Introduction

Electronic cigarette (e-cigarette) use among youth is an emerging public health challenge (1). The industry is growing rapid globally, providing a wide variety of products and attractive flavors actively marketed to children and young adults (2). As a result, there has been a significant increase in the use of e-cigarettes and similar products among youth and adolescents since they entered the Chinese market in 2003 and the European and American markets in the mid-2000s, surpassing the usage rates among adults in many countries (3, 4). Young individuals are particularly prone to being addicted to nicotine. Nicotine addiction may have a detrimental impact on their brain development and mental health (5). Growing evidence suggests that these products have a negative impact on public health (6). Adolescents who develop a nicotine addiction are more likely to continue using tobacco throughout their lives than their never nicotine-dependent peers (1, 3). Adolescents are not only prone to physical dependence, but they are also vulnerable to social and environmental factors that encourage the use of electronic cigarettes (3, 7). The product design, variety of flavors, marketing strategies, and perception of safety and acceptability have significantly enhanced the attractiveness of electronic cigarettes among young individuals, thus resulting in the emergence of new generations who are addicted to nicotine (3, 7). Furthermore, accumulating data shows that electronic cigarettes among children and adolescents act as a precursor to cigarette smoking (8, 9). This fact known as the gateway effect of e-cigarettes for nicotine dependence and cigarette smoking is of particular importance, as the extensive body of literature provides unequivocal evidence that tobacco use in the form of cigarette smoking is the leading preventable cause of premature death and the major causal factor for ischemic heart disease, stroke, lung cancer, COPD and numerous other tobacco-related diseases that affect almost every organ in the human body (4, 10).

The global status of electronic cigarettes and electronic nicotine delivery systems sales bans and regulations differ in various jurisdictions. Some of them ban the sale of e-cigarettes, some allow sale of e-cigarettes but regulate their sale and distribution, some countries regulate nicotine and/or other contents of e-cigarettes, while some countries ban the use of flavors (11). In Turkey, the sale of e-cigarettes is banned since imports are allowed only in small quantities, and production is forbidden (12). E-cigarettes are nevertheless widely available.

E-cigarette use prevalence among university students varies across different countries, possibly as a result of the country’s tobacco and e-cigarette control measures (13–17). More information regarding the use of electronic cigarettes among medical trainees needs to be provided (13, 15). Electronic cigarette use in medical students is particularly concerning because the use of these devices could affect the attitudes, beliefs, and professional behavior of future physicians.

The primary aim of our study was to evaluate the prevalence and predictors of electronic cigarette use among medical faculty students in a country that prohibits the sale of e-cigarettes and their liquids. Additionally, we aimed to assess tobacco use prevalence and its co-occurrence with e-cigarettes among medical trainees. Our study also sought insights into attitudes and beliefs surrounding using electronic cigarettes, which can have significant implications for patient care and medical education policy. By better understanding e-cigarette use among our student population, we can enhance our prevention efforts and optimize the efficacy of our educational and clinical programs.

## 2 Material methods

### 2.1 Study design and study population

The cross-sectional online survey was administered from November 2023 to February 2024. Students from Bursa Uludağ University Faculty of Medicine met the requirements. An email, including a survey developed using the Google Forms website, was sent to all medical faculty students email addresses. Additionally, we posted the survey link on the university’s student affairs webpage, making it accessible only to the university students community. The students willing to participate in the study individually and anonymously filled out the questionnaire. The Clinical Research Ethical Committee of Bursa Uludağ University’s Faculty of Medicine approved the study protocol (approval number: 2023-19/ 1). This study is conducted by adhering to the Helsinki Declaration and upholding ethical norms.

### 2.2 Study questionnaire

The study questionnaire (https://docs.google.com/forms/d/13Nf88_YVJsE0TB2JrUAl4UDiWednic3JpNBa-t9gcDU/edit) included 54 items based on previous studies (16, 18). Questions addressed socio-demographic data such as sex, age, marital status, state of origin, monthly income, living place, academic grade of the school (from 1st year to 6th grade), and students’ parents’ educational level. Educational level was categorized as primary, middle, high school, or university graduate. Monthly income was categorized into three levels: less than 20.000 TRY/month (~ 620 USD/month), 20–40.000 TRY/month, and higher than 40.000 TRY/month (~ 1,240 USD/month). Participants self-reported if they have a chronic disease or medication use.

All of the participants were questioned as to whether they were current smokers, ever or never smokers. An individual who has smoked at least 100 cigarettes throughout their lifetime and has smoked in the past 30 days is categorized as a “current smoker,” whereas someone who has consumed at least 100 cigarettes throughout their lifetime but has not smoked within the past 30 days is classified as a “ex-smoker.” An “ever-smoker” refers to an individual who has smoked at least 100 cigarettes throughout their life, whereas a “never-smoker” is someone who has smoked fewer than 100 cigarettes and does not smoke now. The same categories were used to define water pipe smoking (defined as having even one or two puffs throughout their lifetime for ever-smoking water-pipe, whether it was smoked in the past 30 days as current-smoking, or not having used it within the last 30 days as ex-smoking), roll-your-own (RYO) cigarettes, and cigars (defined as consuming over 100 cigars in their lifetime to be classified as ever-smokers) as well. The age of smoking initiation was also recorded.

Study participants who had ever used electronic cigarettes even one or two times were referred to as “e-cigarette ever-users,” while those who reported current use in the past 30 days were classified as “e-cigarette current users.” Participants who had used electronic cigarettes in the past but not in the past 30 days were defined as “e-cigarette ex-users,” and those who had never used electronic cigarettes (even one or two times) were categorized as “e-cigarette never users.” For e-cigarette users, their consumption patterns were assessed by determining the type of e-cigarette they used (disposable, pre-filled cartridge, or refillable tank), their preferred flavor choice (fruit/mint or menthol/bubble gum/cinnamon/chocolate/other), total amount of cartridge use in milliliters for refillable devices per month, the number of days e-cigarettes were used in the past 30 days, and the age at which they began using e-cigarettes. Dual smoking refers to the simultaneous use of e-cigarettes and conventional cigarettes.

Smoking prevalence and e-cigarette use of students’ parents and their close friends were assessed. The survey investigated students’ understanding of the components of electronic cigarettes, the risks associated with electronic cigarettes, and the hazards of nicotine. The questionnaire also explored students’ knowledge about the ingredients of electronic cigarettes, the hazards of electronic cigarettes, and the nicotine itself. The questionnaire also included the question “Which one do you think is more hazardous to health, electronic cigarettes or cigarettes? “Do you think that electronic cigarettes may aid smoking cessation?,” Do you believe that the flavors in electronic cigarettes are dangerous?,” “Do you believe that second-hand exposure to electronic cigarette vapor is hazardous?”

### 2.3 Statistical analysis

**Figure 1:**
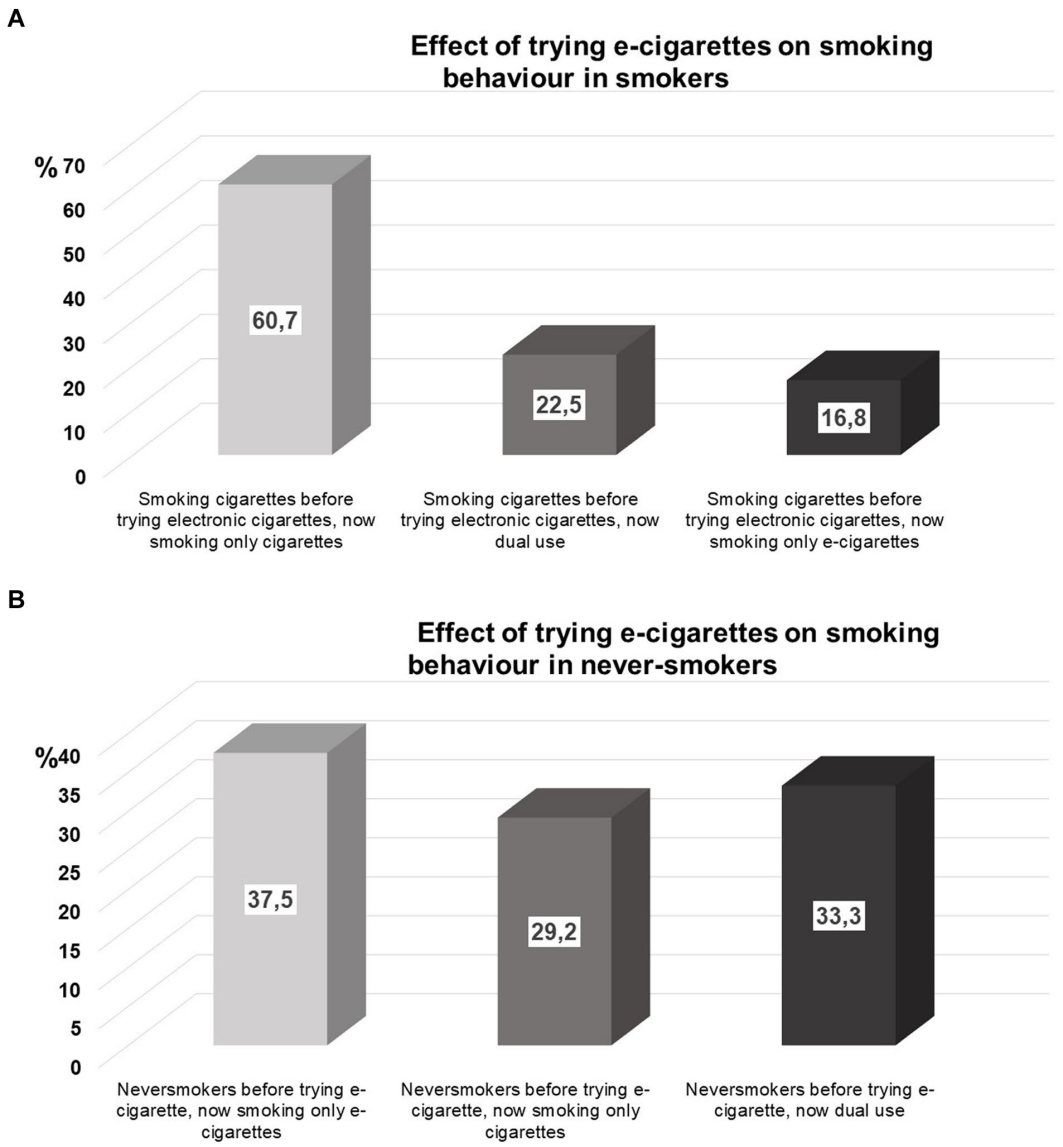
**(A)** Effect of trying e-cigarettes on smoking behavior in smokers, **(B)** Effect of trying e-cigarettes on smoking behavior in never-smokers.

**Figure 2:**
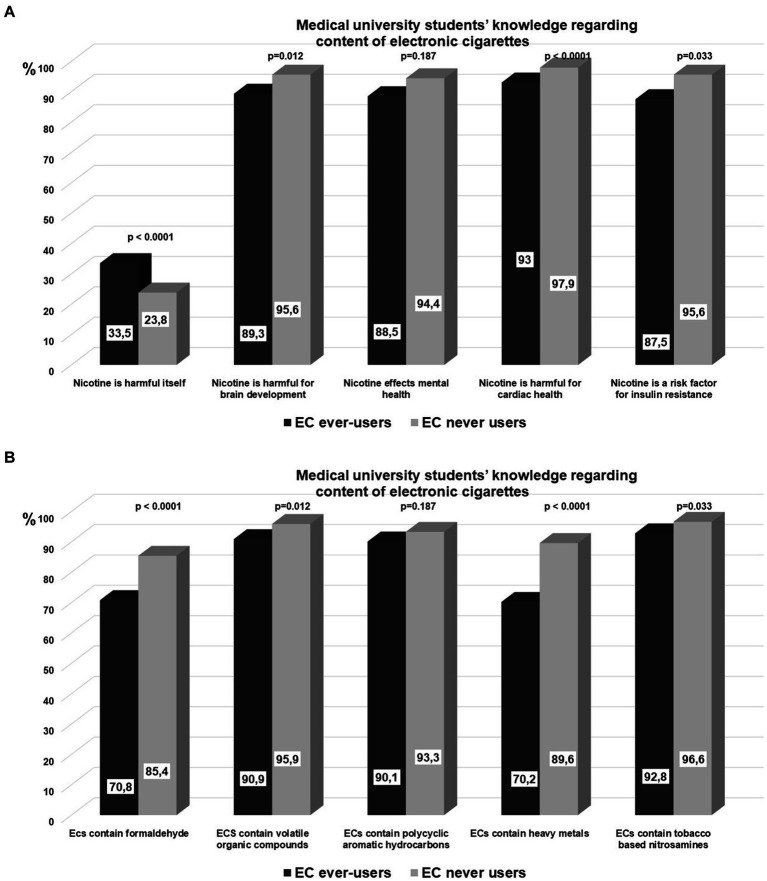
**(A)** Medical university students’ knowledge regarding adverse effects of nicotine. **(B)** Medical university students’ knowledge regarding the content of electronic cigarettes.

The variables were examined using histograms, probability plots, and analytical approaches (such as the Kolmogorov–Smirnov and Shapiro–Wilk’s tests) to ascertain their normal distribution. Continuous variables were represented using means and standard deviations for normally distributed data. The amount of cartridge use in milliliters per month was represented by medians and interquartile ranges (IQR) 25 to 75 as it did not follow a normal distribution. Categorical variables were defined using proportions (Tables 1–5; Figures 1, 2). The Pearson chi-square test or Fisher’s exact test, where appropriate, was used to compare proportions between e-cigarette ever-users and never users as well as dual smokers and others. The Bonferroni test was used for pairwise comparisons (Tables 3–5). The Student’s *t*-test was used to compare continuous outcome variables (Table 3). To maintain a total type I error rate of 5%, *p*-values below 0.05 were considered statistically significant. Binary logistic regression models were utilized to determine the independent predictors (age, sex, monthly income, living place, parental smoking status, close friend’s smoking status, and the presence of a mental disorder) of electronic cigarette ever-use and dual smoking use (the dependent variables in separate models) (Tables 6, 7). Covariates were incorporated into the model if their *p*-value was below 0.10. *p*-values below 0.05 were deemed statistically significant. Statistical analysis was conducted using IBM SPSS Statistics for Windows, Version 28.0 (IBM Corp. Released 2021. IBM SPSS Statistics for Windows, Version 28.0. Armonk, NY: IBM Corp.).

## 3 Results

### 3.1 Study population

A total of 1,054 medical students with a mean age of 21.5 ± 2.6 years old participated in this survey. Total response rate was 54.2%. The female population comprised 51.1% of the study sample. The participants’ sociodemographic features are summarized in Table 1. 16% of the study population reported a physician-diagnosed medical comorbidity. Allergic disorders or asthma and mental disorders (depression and anxiety) were the most frequently reported disorders. Most of the participants were single, and 33.7% of the study population was in the first year of school (Table 1).

**Table 1:** Study participants’ sociodemographic characteristics (*N* = 1,054).

	*N* (%)
Sex	
Males	515 (48.9)
Females	539 (51.1)
Age, years old	21.5 ± 2.6
Marital status^α^	
Single	1,033 (98.0)
Married	18 (1.7)
Academic grade	
1st	355 (33.7)
2nd	169 (16.0)
3rd	143 (13.6)
4th	164 (15.6)
5th	107 (10.2)
6th	116 (11.0)
Country	
Turkey	969 (91.9)
Abroad	85 (8.1)
Medical comorbidities^δ^	
Allergic disorders and asthma	30 (2.9)
Mental disorders (depression and anxiety)	41 (4.0)
Monthly income^ε^	
< 20.000 TRY	311 (30.2)
20.000–40.000 TRY	364 (35.3)
> 40.000 TRY	356 (34.5)
Living place^α^	
Home with family	322 (30.6)
Student dormitory	450 (42.8)
Home with friends/alone	279 (26.5)
Mother educational status^ϕ^	
Primary-school	251 (24.0)
Secondary-school	129 (12.3)
High-school	263 (25.1)
University	405 (38.6)
Father educational status^β^	
Primary-school	129 (12.4)
Secondary-school	106 (10.2)
High-school	251 (24.1)
University	555 (53.3)

Descriptive statistics were given mean ± standard deviation or frequency (*n*) with percentage (%). *N*, number of participants; TRY, Turkish Liras ^α^Data available for 1,051 participants; ^δ^Data available for 1,027 participants; ^ε^ Data available for 1,031 participants; ^ϕ^Data available for 1,048 participants; ^β^Data available for 1,041 participants.

### 3.2 Smoking status

In the study population, the prevalence of current smoking was 17.0% for cigarettes, 2.4% for roll-your-own cigarettes, 4.5% for water pipes, and 3.0% for cigars (Table 2). The rates of ever smoking were 37.7% for cigarettes, 16.0% for roll-your-own cigarettes, 23.6% for water pipes, and 16.8% for cigars.

**Table 2:** Cigarette smoking and e-cigarette use of participants’ parents and close friends.

	Current-use	Ex-use	Never use	*N*
Cigarette	175 (17.0)	213 (20.7)	642 (62.3)	1,030
E-cigarette	41 (4.0)	174 (16.9)	812 (79.1)	1,027
Water pipe	45 (4.5)	189 (19.1)	756 (76.4)	990
RYO cigarette	24 (2.4)	134 (13.6)	829 (84.0)	987
Cigar	30 (3.0)	136 (13.8)	821 (83.2)	987
Mother, cigarette	169 (16.3)	125 (12.1)	740 (71.6)	1,034
Father, cigarette	352 (34.7)	271 (26.7)	391 (38.6)	1,014
Close friend, cigarette	388 (37.9)	50 (4.9)	587 (57.2)	1020
Mother, e-cigarette	6 (0.6)	–	1,048 (99.4)	1,054
Father, e-cigarette	8 (0.8)	10 (0,9)	1,036 (98.3)	1,054
Close friend, e-cigarette	114 (20.9)	23 (4.2)	408 (74.9)	545

Data is presented as frequency (*n*) with percentage (%). *N*, number of participants; RYO, roll-your-own.

### 3.3 Parents’ sociocultural level and parental smoking

When questioned regarding their accommodations, most of the students were found to be residing in a student dormitory, followed by a significant number remaining with their families, and ~ 1/4 staying at home with friends (Table 1). The monthly income of students’ families was divided into three categories, with each category nearly representing one-third of the students (Table 1). Of the overall study participants, 24.0% of mothers and 12.4% of fathers had completed primary school, while 38.6% of mothers and 53.3% of fathers had a university-level education (Table 1). The prevalence of current smoking among students’ mothers and fathers was 16.3 and 34.7% for cigarette smoking, whereas 0.6 and 0.8% for e-cigarette current use, respectively (Table 2).

### 3.4 E-cigarette use

Out of 1,027 respondents, 215 individuals (20.9%) reported ever-use of electronic cigarettes or similar products at least once. 41 out of 1,027 individuals (4.0%) indicated regular use of e-cigarettes or heat-not-burn tobacco products, whereas 16.9% reported ex-use. 79.1% had never used them (Table 2). The mean age for starting e-cigarettes was reported as 20.0 ± 3.0 years old. The average duration of e-cigarette use in the past 30 days was 4.2 ± 8.8 days for ever-users. The median refillable cartridge monthly usage was 40 mL, with an interquartile range (IQR 25–75) of 0.5 mL to 90.0 mL (data not shown). We observed that the mean age of smoking initiation was younger than e-smoking initiation (17.7 ± 6.0 vs. 20.0 ± 3.0 years old, *p* < 0.0001, data not shown).

### 3.5 Consumption patterns

We observed that of those e-cigarette ever-users, 49.3% used disposable e-cigarettes, 32.5% used refillable tank-style devices, and 18.2% used replaceable pre-filled cartridges. All e-cigarette users were using flavored e-cigarettes. Among the ever-users surveyed, the most popular flavor types were fruit (67.2%), mint or menthol (19.0%), cinnamon (5.7%), bubblegum (5.2%), and chocolate (2.9%). 56.4% of the users obtained e-cigarettes from tobacco shops, whereas 25.4% from online trade, 9.1% from peers and friends, 5.4% from abroad or airports, and 3.6% from social media.

When we questioned the effect of trying e-cigarette use for the first time on participants following smoking habits, 113 out of 215 e-cigarette ever-users answered this item. We observed that (*n* = 89, 78.8%) of the students who experimented with e-cigarettes were already smoking cigarettes. Out of the initial group of cigarette smokers (78.8%), 22.5% started using both cigarettes and e-cigarettes, while 60.7% kept smoking cigarettes only, and 16.8% exclusively used e-cigarettes, as shown in Figure 1A. Conversely, 22.2% of the students (*n* = 24) who used e-cigarettes had never smoked before. Nevertheless, after experimenting e-cigarettes, 37.5% of individuals persisted in using e-cigarettes, 33.3% participated in both e-cigarette and traditional cigarette use, and 29.2% initiated smoking cigarettes, as seen in Figure 1B.

### 3.6 The reasons for using electronic cigarettes and complaints related with electronic cigarettes

The main reasons for using e-cigarettes among ever-users were curiosity (42.7%), better taste than cigarettes (25.0%), their intent to quit smoking (16.1%) or to reduce cigarette smoking (11.3%), and their belief that e-cigarettes are less harmful to the environment (8.9%). The main complaints that started after using electronic cigarettes were cough (58.4%), dyspnea (54.2%), sore throat (41.3%), dizziness (23.4%), anxiousness (15.9%), teeth and gingiva problems (18.7%), gastrointestinal problems (17.8%), and polyuria (9.3%) among study participants.

### 3.7 Correlates of ever using e-cigarettes among medical faculty students

Males were more likely to use e-cigarettes than females (*p* < 0.001, Table 3). Students who never used e-cigarettes were younger than ever-users (*p* = 0.038). E-cigarette ever-users had 6.5 percent mental comorbidities, compared to 3.3% for never-smokers (*p* = 0.048). There were more e-cigarette ever-users (42.9%) than non-users (32.9%) in the highest monthly income category (Table 3). More students who used e-cigarettes (33.2%) stayed home with friends or alone than those who never did (24.8%, *p* = 0.086). No difference in e-cigarette use was found across academic years (Table 3).

**Table 3:** Characteristics of electronic cigarette ever-users and never users and dual users.

	Electronic cigarette			Dual smoking		
	Ever-user	Never user	*p* value	Dual smoker	Either	*p* value
Sex, %						
Males	34.4^a^	55.8^b^	<0.001	67.9^a^	46.4^b^	<0.001
Females	65.6^a^	44.2^b^		32.1^a^	53.6^b^	
Age, years old	21.6 ± 3.0	21.1 ± 3.3	0.038	21.7 ± 2.5	21.1 ± 3.2	0.072
Marital status, %						
Single	97.7	98.4	0.556	97.3	98.4	0.007
Married	2.3	1.6		2.7	1.6	
Academic grade, %						
1st	27.1	35.3	0.194	23.2	35	0.077
2nd	16.8	15.5		17	15.7	
3rd	12.1	13.6		13.4	13.3	
4th	18.7	15.2		20.5	15.1	
5th	11.2	9.9		8,9	10.5	
6th	14	10.4		17	10.5	
Country of residence, %						
Turkey	92.1	93.4	0.543	96.4	92.6	0.168
Abroad	7.9	6.6		3.6	7.4	
Medical comorbidities, %						
Allergic disorders and asthma	11.2	8.1	0.175	10.7	8.4	0.377
Mental disorders (depression and anxiety)	6.5	3.3	0.048	1.8	1.1	0.378
Monthly income, %						
< 20.000 TRY	25.2	30.7	0.025	26.8	30.4	0.036
20.000–40.000 TRY	31.9	36.4		27.7	36.2	
> 40.000 TRY	42.9^a^	32.9^b^		45.5^a^	33.4^b^	
Living place, %						
Home with family	26.6	32.2	0.086	24.3	31.4	0.133
Student dormitory	40.2	43		42.3	42.8	
Home with friends/alone	33.2^a^	24.8^b^		33.3	25.8	
Mother, cigarette smoking, %						
Current smoker	26.1^a^	14.2^b^	<0.001	26.6^a^	15.3^b^	0.007
Ex-smoker	10.9	12.5		13.8^a^	12.0^b^	
Never-smoker	63.0^a^	73.3^b^		59.6^a^	72.6^b^	
Father, cigarette smoking, %						
Current smoker	45.0^a^	31.9^b^	<0.001	54.5^a^	32.3^b^	<0.001
Ex-smoker	25.1	27.5		21.8^a^	27.4^b^	
Never-smoker	29.9^a^	40.6^b^		23.6	40.3	
Close friend, cigarette smoking, %						
Current smoker	71.6^a^	29.0^b^	<0.001	83.6^a^	32.0^b^	<0.001
Ex-smoker	4.7	5.1		2.7^a^	5.2^b^	
Never-smoker	23.7^a^	65.9^b^		13.6	62.8	
Mother, e-cigarette smoking, %						
Current smoker	1.9^a^	0.2^b^	0.02	1.8	0.4	0.129
Ex-smoker	-	-		-	-	
Never-smoker	98.1^a^	99.8^b^		98.2	99.6	
Father, e-cigarette smoking, %						
Current smoker	2.3^a^	0.4^b^	0.015	3.6^a^	0.4^b^	0.002
Ex-smoker	0.9	1		0.9^a^	1.0^b^	
Never-smoker	96.7	98.6		95.5	98.6	
Close friend, e-cigarette smoking, %						
Current smoker	54.7^a^	12.0^b^	<0.001	67.2^a^	16.7^b^	<0.001
Ex-smoker	6.8	3.3		9.8^a^	3.5^b^	
Never-smoker	38.5^a^	84.7^b^		27.5^a^	79.8^b^	

Descriptive statistics were given mean ± standard deviation or as percentage (%). Column percentages are reported. Pairwise comparisons were conducted using the Bonferroni test. The superscripts “a” and “b” indicate the results of pairwise comparisons across groups. Values with different letters indicate significant differences between groups. TRY, Turkish Liras.

E-cigarette ever-users whose mothers smoke were more common than non-users (*p* < 0.001). Similar results were found for e-cigarette users’ fathers who smoke (45.0% vs. 31.9%, *p* < 0.001). Similarly, the prevalence of e-cigarette use among both mothers (1.9%) and fathers (2.3%) who have ever used e-cigarettes was higher compared to those who have never used e-cigarettes (Table 3). E-smoking parents had similar educational backgrounds to non-smokers (data not shown). Close friends’ present smoking rate was greater in e-cigarette ever-users (71.6%) than never users (29.0%), while their never smoking rate was significantly lower (23.7%) than 65.9%, see Table 3.

After adjusting for potential confounders, multivariable analysis showed that male medical students had a higher likelihood of using e-cigarettes compared to females (adjusted OR: 1.85; 95% CI: 1.30–2.62; *p* < 0.001). Students who had the highest monthly income were more likely to have used e-cigarettes (adjusted OR: 1.60; 95% CI: 1.04–2.46; *p* = 0.035). We also observed that medical students whose friends were currently smoking cigarettes were more likely to ever use e-cigarettes (adjusted OR: 5.54; 95% CI: 3.81–8.06; *p* < 0.001), see Table 4.

**Table 4:** Associations of electronic cigarette ever-use and dual smoking.

	Electronic cigarette ever-use			Dual smoking		
	OR	(95% CI)	*p* value	OR	(95% CI)	*p* value
Sex						
Female	Ref			Ref		
Male	1.85	1.30–2.62	<0.001	1.81	1.14–2.87	0.012
Age	1.01	0.96–1.06	0.713	1.02	0.96–1.09	0.486
Monthly income						
< 20.000 TRY	Ref			Ref		
20.000–40.000 TRY	1.05	0.67–1.63	0.835	0.88	0.49–1.60	0.683
> 40.000 TRY	1.6	1.04–2.46	0.035	1.72	0.98–3.01	0.058
Living place						
Home with family	Ref			Ref		
Student dormitory	1.05	0.68–1.62	0.819	1.28	0.72–2.28	0.396
Home with friends/alone	0.99	0.64–1.55	0.996	0.98	0.55–1.73	0.935
Mother, cigarette smoking						
Never smoker	Ref			Ref		
Ever smoker	1.25	0.86–1.82	0.237	1.28	0.80–2.05	0.303
Father, cigarette smoking						
Never smoker	Ref			Ref		
Ever smoker	1.29	0.89–1.89	0.177	1.79	1.06–3.00	0.027
Close friend, cigarette smoking						
Never smoker	Ref			Ref		
Ever smoker	5.54	3.81–8.06	<0.001	9.85	3.35–18.12	<0.001
Mental disorders	2.02	0.96–4.28	0.065	1.66	0.66–4.21	0.285

Ref, reference category; TRY, Turkish Liras; OR, odds ratio; CI, confidence intervals.

### 3.8 Dual smoking

Current cigarette, water pipe, RYO cigarette, and cigar smoking prevalence rates were 52.3, 16.1, 9.0, and 12.0% among electronic cigarette ever-user students (Table 5). All those prevalence rates were significantly higher than students who currently smoke cigarettes, water pipes, RYO cigarettes, or cigars but never used e-cigarettes (7.6, 1.4, 0.6, and 0.6%, respectively). We also observed that e-cigarette ever-use prevalence among ex – cigarette, water pipe, RYO cigarette, and cigar smokers were significantly higher (40.2, 57.8, 48.3, and 46.0%, respectively) than that of students who quit cigarettes, water pipes, RYO cigarettes, cigars and never used e-cigarettes (15.4, 9.4, 4.5, and 5.6%, respectively), see Table 5.

**Table 5:** Prevalence of dual smoking in electronic cigarette ever-users and never users.

	Electronic cigarette		
	Ever-user	Never user	*p* value
	*N* (%)	*N* (%)	
Cigarette smoking^α^			
Current smoker	112 (52.3)^a^	61 (7.6)^b^	<0.001
Ex-smoker	86 (40.2)^a^	123 (15.4)^b^	
Never-smoker	16 (7.5)^a^	617(77.0)^b^	
Water pipe smoking^β^			
Current smoker	32 (16.1)^a^	11 (1.4)^b^	<0.001
Ex-smoker	115 (57.8)^a^	73 (9.4)^b^	
Never-smoker	52 (26.1)^a^	693 (89.2)^b^	
RYO cigarette smoking^δ^			
Current smoker	18 (9.0)^a^	5 (0.6)^b^	<0.001
Ex-smoker	97 (48.3)^a^	35 (4.5)^b^	
Never-smoker	86 (42.8)^a^	733 (94.8)^b^	
Cigar smoking^δ^			
Current smoker	24 (12.0)^a^	5 (0.6)^b^	<0.001
Ex-smoker	92 (46.0)^a^	43 (5.6)^b^	
Never-smoker	84 (42.0)^a^	726 (93.8)^b^	

Descriptive statistics were given mean ± standard deviation or frequency (*n*) with percentage (%). Column percentages are reported. Pairwise comparisons were conducted using the Bonferroni test. The superscripts “a” and “b” indicate the results of pairwise comparisons across groups. Values with different letters indicate significant differences between groups. N, number of participants; RYO, roll-your-own ^α^ Data available for 1,015 participants ^β^ Data available for 976 participants ^δ^ Data available for 974 participants.

After controlling for sex and age, we observed similar associations with e-cigarette ever-use for dual smoking in our multivariable models. Students who smoked cigarettes, water pipes, or cigars were 15.29, 4.02, and 4.32 times more likely to use e-cigarettes (Table 6). Additionally, students who quit smoking were 10.65 times (95 CI, 5.54–20.49) more likely to use e-cigarettes. Students who quit water pipe, RYO cigarette, and cigar smoking were 4.39 times, 2.39 times, and 2.07 times more likely to use e-cigarettes (Table 6). Importantly, we found that male sex (OR: 1.81; 95% CI: 1.14–2.87; *p* = 0.012), both fathers’ (OR: 1.79; 95% CI: 1.06–3.00; *p* = 0.027) and close friends’ current smoking status (OR: 9.85; 95% CI: 3.35–18.12; *p* < 0.001) were independent risk factors for dual smoking (Table 4).

**Table 6:** Associations between electronic cigarette ever-use and smoking other tobacco products.

	Electronic cigarette ever-use		*p* value
	OR	(95% CI)	
Cigarette smoking			
Current smoker	15.29	7.18–32.58	<0.001
Ex-smoker	10.65	5.54–20.49	<0.001
Never-smoker	Ref	–	
Water pipe smoking			
Current smoker	4.02	1.51–10.72	0.005
Ex-smoker	4.39	2.57–7.51	<0.001
Never-smoker	Ref	–	
RYO cigarette smoking			
Current smoker	1.59	0.45–5.66	0.473
Ex-smoker	2.39	1.31–4.33	0.004
Never-smoker	Ref	–	
Cigar smoking			
Current smoker	4.32	1.26–14.79	0.02
Ex-smoker	2.07	1.15–3.72	0.015
Never-smoker	Ref	–	

Models were controlled for sex, age. For tobacco products, never-smoking individuals were accepted as the reference category. Ref, reference category; RYO, roll-your-own; OR, odds ratio.

### 3.9 Knowledge about and perspectives on electronic cigarettes

Of the 858 respondents over 1,054, 20.7% (*n* = 178) thought e-cigarettes were much more harmful, 13.9% (*n* = 119) slightly more harmful than regular cigarettes, and 39.4% (*n* = 338) thought that e-cigarettes were as harmful as regular cigarettes. On the other hand, 17.4% (*n* = 149) believed that e-cigarettes were slightly less harmful, while 8.6% (*n* = 74) believed they were much less harmful than regular cigarettes (data not shown). 81.6 percent agreed e-cigarettes are addictive. E-cigarette vapor was exposed to 54.4% of study participants, whereas 6.7% used them in public. 67.7% stated second-hand e-cigarette smoke was harmful. 13.2% of participants thought e-cigarettes may help in quit smoking, 52.9% disagreed, and 33.9% were neutral (data not displayed).

Our analyses of medical faculty students’ perceptions of electronic cigarettes are summarized in Table 7. More electronic cigarette users (41.5% vs. 21.5%, *p* < 0.0001) feel e-cigarettes are less dangerous than regular cigarettes and aid in smoking cessation (29.3% vs. 8.9%, *p* < 0.0001). Not surprisingly, more e-cigarette ever-users agreed using e-cigarettes indoors than never users (27.6% vs. 0.3%, *p* < 0.0001). E-cigarette ever-users were less likely to perceive second-hand smoking vapor as harmful (68.7% vs. 83.6%, *p* < 0.0001) than never users. More e-cigarette consumers (10.2% vs. 2.2%, *p* < 0.0001) disagreed that e-cigarette flavors may be harmful to health. E-cigarette users were more likely to disagree that e-cigarettes have higher nicotine concentration than conventional cigarettes (22.1% vs. 6.9%, *p* < 0.0001). Figure 2A shows that the information level about the adverse health effects of nicotine was lower in e-cigarette ever-users compared to never users. Similarly, e-cigarette users were less aware than non-users that e-cigarettes include heavy metals, tobacco-based nitrosamines, polycyclic aromatic hydrocarbons, formaldehyde, and volatile organic compounds, Figure 2B.

**Table 7:** Students perspectives on electronic cigarettes.

	Electronic cigarettes		
	Ever-user %	Never user %	*p* value
Which suggestion do you agree?			
ECs are less harmful than cigarettes	41.4^a^	21.5^b^	<0.0001
ECs are as harmful as cigarettes	31.6^a^	41.7^b^	
ECs are more harmful than cigarettes	27.4^a^	36.9^b^	
ECs are helpful for smoking cessation			
Agree	29.3^a^	8.9^b^	<0.0001
Disagree	49.7	53.7	
Neutral	20.9^a^	37.4^b^	
Do you agree using ECs indoors?			
Agree	27.6^a^	0.3^b^	<0.0001
Disagree	65.8^a^	98.5^b^	
Neutral	6.6^a^	1.2^b^	
Do you think second-hand smoking ECs vapor is harmful?			
Agree	68.7^a^	83.6^b^	<0.0001
Disagree	21.2^a^	12.3^b^	
Neutral	10.1^a^	4.1^b^	
Do you think flavors in ECs vapor is harmful?			
Agree	67.0	69.4	<0.0001
Disagree	10.2^a^	2.2 ^b^	
Neutral	22.8	28.4	
Do you think nicotine concentration in ECs is higher than cigarettes?			
Agree	37.9	37.8	<0.0001
Disagree	22.1^a^	6.9^b^	
Neutral	40.0^a^	55.3^b^	

Descriptive statistics were given as column percentage (%). Pairwise comparisons were conducted using the Bonferroni test. The superscripts “a” and “b” indicate the results of pairwise comparisons across groups. Values with different letters indicate significant differences between groups. Definitions of abbreviations: N, numbers; EC, electronic cigarettes.

## 4 Discussion

Results of this study show that cigarette smoking is the most prevalent form of tobacco consumption among medical university students of Bursa Uludağ University in Turkey, with a current smoking rate of 17.0% and an ever-smoking prevalence of 37.7%. Although sales of electronic cigarettes and e-liquids are prohibited in Turkey, the prevalence of e-cigarette ever-use was as high as 20.9%. The current e-cigarette use prevalence was 4.0%. A remarkable 52.3% of e-cigarette users also smoke cigarettes. Moreover, 16.1% of individuals who use e-cigarettes concurrently smoke water pipes, demonstrating substantial poly tobacco use. Correlates of ever-using e-cigarettes among medical faculty students were being male, having a higher monthly income, and having a current smoker friend. Male sex, paternal current smoking, and close friends’ current smoking status were independent risk factors for dual smoking. More students who use e-cigarettes believe they are less harmful than cigarettes and may help them quit smoking. Additionally, more electronic cigarette users seem to underestimate the toxic substances in e-cigarettes and nicotine’s health risks.

It is important to note the high and significant smoking prevalence rates among medical university students in our study. According to the Global Adult Tobacco Survey in 2016, the prevalence of current cigarette smoking among individuals aged 15 and over in Turkey was 31.6% (19). Subsequent data from the Health Interview Survey in 2019 indicated that the overall prevalence of tobacco smoking in Turkey was 31.3%, suggesting that smoking rates remained high in the country during this period (20). In comparison, smoking prevalence in Turkey is notably higher than in Europe (25.9% in 2017) and the United States (10.8% in 2023) (21–23). When considering university students’ age group, survey data indicates very high smoking rates among university students in various countries. A survey conducted between 2017 and 2018, which included 14,352 university students from Belarus, Lithuania, Poland, Russia and Slovakia (the sales of e-cigarettes are allowed with regulation) reported very high smoking rates. The survey revealed that 66.1% of the study population were ever cigarette users, with over two thirds (68.9%) of medical university students were ever cigarette smokers (17). Furthermore, another large international study enrolling 7,526 medical university students from Brazil (bans sales of e-cigarettes), United States (allow sales of e-cigarettes but regulate) and India (bans sales of e-cigarettes) between 2020 and 2021, showed remarkably high tobacco smoking rates, with 31.7% in students from Brazil, 50.1% in students from U.S, and 7.1% in students from India (13). Some of these countries have legislation that permit the sale of e-cigarettes, while others have outright bans on their sale. Based on these findings, we believe that cigarette smoking rates are elevated in numerous countries, irrespective of their e-cigarette policies.

This study found that 20.9% of medical students had ever used e-cigarettes, with a 4.0% current use rate. In comparison, data from 2015–2018 Global Adult Tobacco Survey showed that 2.2% of young adults and adults over 15 had ever used e-cigarettes in Turkey, while 1.3% reported current use (24). In 2019, the cigarette smoking prevalence was 38.4% and regular e-cigarette use was 3.5% in our university students, which was a substantial rise compared to earlier data (18, 24, 25). Over the 4-year period, there was a small decline (from 38.4 to 37.7%) in the prevalence of ever-smoking among medical university students and a slight increase in the regular use of electronic cigarettes (from 3.5 to 4.0%). These findings may suggest a shift towards new-generation tobacco products in medical university students in Turkey.

When compared to studies enrolling university students reported from several other countries, e-cigarette use among university students in Turkey is lower compared to some countries such as Brazil (19.8% current use among medical trainees in 2021), the U.S. (10.9% current use among medical trainees in 2021), China (32.4% ever-use among university students in 2020), New Zealand (40.5% ever-use and 6.1% current use in 2018), Poland (8.6% current use among medical trainees in 2020), Belarus (42.7% ever-use among university students between 2017–2018), Lithuania (56.6% ever-use among university students between 2017–2018), Poland (45.0% ever-use among university students between 2017–2018), Russia (33.4% ever-use among university students between 2017–2018), Slovakia (34.4% ever-use among university students between 2017–2018), Jordan (10.5% among university students, between 2020 and 2021) and Saudi Arabia (11.5% among medical students in 2019) (13, 15, 17, 26–28). However, comparing these studies is challenging due to differences in study designs, populations, time intervals, definitions of smoking outcomes, and different regulations in each country for e-cigarette sales and import. The prevalence of e-cigarette use found in our study (4.0% current use and 20.9% ever-use) is consistent with the results of a limited number of studies performed in Turkey among the young population (29–31). These findings suggest that governments should implement evidence-based, strong tobacco control programs to protect university students from smoking or using any tobacco or nicotine products that may contribute to nicotine addiction. These initiatives should be consistent with the WHO Framework Convention on Tobacco Control for member nations (32).

The import of e-cigarettes and similar tobacco products was prohibited in 2020 in Turkey. As of July 2021, no e-cigarette has been officially approved for sale in Turkey (12). E-cigarette use in Turkey remains less common compared to several other countries. As Glantz highlighted, the prevalence of e-cigarette usage in Turkey is comparable to countries like Brazil and Thailand, where the import and sale of e-cigarettes are prohibited (12). Nevertheless, our analysis reveals that the present rate of e-cigarette usage among medical faculty students is more than twice the rates reported in GATS, 2016. This indicates a concerning upward trend despite existing regulations in the country (24). On the other hand, we observed that our students were able to easily access e-cigarettes from tobacco shops (more than 50%) and through online sales (25%), suggesting that more effective preventive measures should be implemented in our country.

Despite differences in vaping prevalence between studies, risk factors identified for vaping were similar among studies. DEBRA study indicated male sex and smoking status as risk variables, while YUPESS identified male sex (16, 17). In a large US and Brazilian medical student population, Degani-Costa and colleagues discovered that vaping was connected with the male sex, increasing household income, and tobacco smoking in the last year (13). The effect of peer cigarette smoking on e-cigarette use in adolescents was shown in large population studies (33–35). We identified male sex, higher monthly income, and having a current smoker friend as correlates of ever-using e-cigarettes among medical faculty students in our study. Most of those studies did not include paternal and peer smoking as covariates in their models of risk factors for e-cigarette use, but we showed that a close friend’s smoking status strongly predicts e-cigarette ever-use.

Dual use was common in our study. 52.3% of e-cigarette users also smoked cigarettes. These findings align with 44–62% dual use rates from earlier research (16, 17, 30). Water pipe was the second most popular tobacco product among e-cigarette users, after cigars and RYO cigarettes (Table 6). Another interesting finding, we observed was that cigarette, water pipe, RYO, and cigar ex-smokers used electronic cigarettes more than students who never used them. Smoking any tobacco product was an independent risk factor for e-cigarette ever use in multivariable models. After controlling for sex and age, ex-smoking cigarettes, water pipes, or cigars were independent risk factors for e-cigarette ever-use, which may suggest a shift in medical school students’ product preferences towards electronic cigarettes (Table 6). Furthermore, our findings demonstrated that peer smoking status is the most influential factor in predicting dual use. The high prevalence of dual use observed in our study, as well as observed in earlier investigations, may indicate a renormalization of smoking habits triggered by electronic cigarettes, even in medical faculty students.

When we analyzed the impact of e-cigarette use on smoking habits, we noted that a significant majority (78.8%) of the students who experimented with e-cigarettes were already regular cigarette smokers, Figure 1A. Of those baseline smokers ~1/4 continued with dual use, whereas 17% continued with using e-cigarettes, Figure 1A. On the other hand, around one-fourth of the students who used e-cigarettes had never smoked before, Figure 1B. Nevertheless, following their experience with e-cigarettes, 29% of individuals began smoking traditional cigarettes, 38% began using electronic cigarettes, and around one-third started dual use (Figure 1). Soneji and colleagues conducted a thorough systematic analysis, which revealed that adolescents and young adults who had previously used e-cigarettes were 3.50 times more likely to initiate cigarette smoking in comparison to individuals who had never used e-cigarettes (9). Leventhal et al. demonstrated that the use of e-cigarettes during early adolescence was linked to an increased probability of using any form of tobacco product during follow-up (8). Hence, our study findings corroborate the notion that electronic cigarettes serve as a pathway for the initiation of smoking, even among students in medical school.

Consistent with prior findings, we observed that curiosity about the novelty of electronic cigarettes, the belief that they are less harmful than traditional cigarettes, and the better taste due to various flavors were the main reasons young people started using them (1, 36). The consumption patterns and product choices of users can vary depending on the availability of products in the local market (13, 16). The primary choice among our students was disposable electronic cigarette types and just flavored ones. The average age of our students who started using electronic cigarettes was 20.0 ± 3.0 years old, similar to previous findings reported between 19 and 21 in medical students from Brazil, India, and the US (13). This age group mainly involves individuals in the university and college phases, highlighting the significance of tobacco control efforts in preventing the initiation of smoking and vaping among university students.

E-cigarette liquids and aerosols contain numerous harmful chemical substances and potential carcinogens such as tobacco-specific nitrosamines, metals (nickel, tin, lead), volatile organic compounds, reactive carbonyls (acetaldehyde, formaldehyde), propylene glycol, glycerol, polycyclic aromatic hydrocarbons, in addition to nicotine and flavoring additives (37–39). The use of e-cigarettes results in measurable exposure to those chemicals and compounds (40, 41). Depending on the flavor, e-cigarette flavorings were cytotoxic in cell-culture models (41–45). Recent findings demonstrate that using e-cigarettes results in detectable exposure to tobacco-related toxicants. However, when compared to those who only use regular cigarettes, biomarker concentrations of nicotine and toxicants among e-cigarette-only users are lower (40). When we examine the knowledge level of our study populations about toxicant ingredients in cigarettes and e-cigarettes, it is evident that even though they are medical students, they possess a limited understanding of the relative quantities of toxicants in regular cigarettes versus e-cigarettes. Although e-cigarettes may have fewer toxicants than regular cigarettes, they nonetheless pose health hazards to users. Additionally, dual use of e-cigarettes with regular cigarettes, which accounts least 50% of the e-cigarette users in this study, is associated with the highest level of toxicant exposure (40).

Nicotine is a highly addictive psychoactive chemical that causes tobacco dependence. Accumulating evidence shows the adverse health effects of nicotine. Nicotine promotes angiogenesis, cell proliferation, epithelial-mesenchymal transition, vascular remodeling and dysfunction, inflammation, and oxidative stress in vascular smooth muscle cells causing atherosclerosis, dendritic cell morphology changes, and neuronal signaling, which may lead to mental disorders like depression, addiction, and attention deficit (46–53). Our study provides comprehensive insights into medical university students’ knowledge of electronic cigarette composition and the health risks associated with nicotine. More than 80% of medical university students are familiar with the constituents of electronic cigarettes, and over 90% were aware of the health risks associated with nicotine. Besides, 26.0 and 13.2% of medical faculty students believe that e-cigarettes are less harmful than regular cigarettes, and can be beneficial for quitting smoking, respectively. A large multinational survey from Brazil, the US, and India, revealed that 27.4 and 32.4% of medical trainees believed e-cigarettes were less harmful than tobacco smoking and endorsed them as a smoking cessation aid (54). These findings suggest that medical university students’ perceptions of e-cigarettes are consistent regardless of the different regulations and bans in various countries and jurisdictions. On the other hand, medical trainees’ perceptions for using e-cigarettes as a smoking cessation aid increases in countries where sales of e-cigarettes are allowed. Medical trainee’s cigarette smoking and e-cigarette use status are also determinant of the perception. We found that almost 40% of medical students who ever used electronic cigarettes thought that e-cigarettes were less harmful than cigarettes, while 30% found e-cigarettes beneficial for quitting smoking. Brozek and colleagues have shown that cigarette smoker, e-cigarette user and dual user university students were 1.83, 15.56, and 10.50 times more likely to believe that e-cigarettes are safe as compared to never smokers (17). Studies have shown, people who smoke or use both conventional and e-cigarettes are more likely to believe e-cigarettes are safe (55, 56). This could be related to the tobacco industry’s powerful marketing techniques that promote electronic cigarettes as safer alternatives to regular cigarette smoking (1, 3). A new study conducted among physicians, primarily pulmonologists, found that smoking cessation training is an independent factor in thinking electronic cigarettes are not helpful for smoking cessation (57).

Our findings are subject to several limitations. First of all, our study included students from a medical university. Due to the inclusion of a specific group, the findings of our study do not reflect the general population. On the other hand, medical trainees are a unique group since they are exposed to thorough information about the health risks of electronic cigarettes as part of their academic curriculum. Furthermore, because medical trainees will be consulting patients in the coming years, their attitudes and knowledge about these products are of particular importance. Secondly, study participants self-reported cigarette, e-cigarette, or other tobacco product use, which may cause an underreported prevalence for use. However, research has shown that biological verification with saliva and urine cotinine levels correlates with self-reported smoking (58). Third, the response rate was rather low compared to the total number of medical faculty students (54.2% response rate), which may limit the generalizability of our findings. Fourth, our survey examined students’ knowledge and opinions about electronic cigarettes. As a result, the findings of this study did not allow us to distinguish between the consumption patterns and preferences of those two product types. On the other hand, there are few reports on the prevalence and views of medical university students toward e-cigarette use in the literature. Thus, our study is one of the studies that report medical trainees’ understanding of e-cigarettes and the risks of nicotine in depth. Furthermore, our country is an interesting population sample because it is one of the few countries that prohibits selling electronic cigarettes.

In conclusion, more than a third of medical students have ever smoked, and 17% currently smoke. Despite Turkish law prohibiting the sale of electronic cigarettes and e-liquids, 20,9% of the medical trainees were able to access and use them. The current use of e-cigarettes has increased from 3.5 to 4.0% in the last four years. A remarkable 52.3% of e-cigarette users smoke cigarettes. Additionally, 16.1% of e-cigarette users smoke water pipes. Male sex, higher income, and close friends’ smoking status were predictors for e-cigarette use among medical university students, while male sex, paternal smoking, and close friends’ smoking status were for dual smoking. More e-cigarette users underestimate the hazardous compounds in e-cigarettes and nicotine’s health risks. More students who use e-cigarettes think they are safer than traditional cigarettes and may help them quit.

## Data Availability

The raw data supporting the conclusions of this article will be made available by the authors, without undue reservation.
